# Gene networks and liar paradoxes

**DOI:** 10.1002/bies.200900072

**Published:** 2009-10

**Authors:** Mark Isalan

**Affiliations:** EMBL-CRG Systems Biology Research Unit, Centre for Genomic Regulation (CRG)UPF, Doctor Aiguader 88, 08003 Barcelona, Spain

**Keywords:** feedback, gene network, liar paradox, oscillator, systems biology

## Abstract

Network motifs are small patterns of connections, found over-represented in gene regulatory networks. An example is the negative feedback loop (*e.g*. factor A represses itself). This opposes its own state so that when ‘on’ it tends towards ‘off’ – and *vice versa*. Here, we argue that such self-opposition, if considered dimensionlessly, is analogous to the liar paradox: ‘This statement is false’. When ‘true’ it implies ‘false’ – and *vice versa*. Such logical constructs have provided philosophical consternation for over 2000 years. Extending the analogy, other network topologies give strikingly varying outputs over different dimensions. For example, the motif ‘A activates B and A. B inhibits A’ can give switches or oscillators with time only, or can lead to Turing-type patterns with both space and time (spots, stripes or waves). It is argued here that the dimensionless form reduces to a variant of ‘The following statement is true. The preceding statement is false’. Thus, merely having a static topological description of a gene network can lead to a liar paradox. Network diagrams are only snapshots of dynamic biological processes and apparent paradoxes can reveal important biological mechanisms that are far from paradoxical when considered explicitly in time and space.

## Introduction

‘The first statement in this paper is false. Therefore, this paper should be rejected’. Although we are hoping for a more sympathetic peer review process, the first sentence raises interesting possibilities: if the sentence is true, it states it is false. If false, its opposite must be true, so it is true. Thus, the outcome of review would be an endless cycle (and, although it may sometimes seem otherwise, real peer review is never like this). Therefore, we have a problem; such self-referential arguments are termed ‘liar paradoxes’ and are attributed either to Epimenides (6th century BC) or Eubulides (4th century BC). The former, a Cretan, is thought to have been the first to create confusion by declaring that ‘all Cretans are liars’.^1^ Although this formulation is actually not a paradox (a resolution is that *some* Cretans are liars), there are stronger formulations, including: ‘The following statement is true. The preceding statement is false’.

This essay aims to develop the analogy that we recently proposed between biological network connections and liar paradoxes.^2^ It is extremely common to find self-opposing biological interactions, resulting in autoregulation or negative feedback.^3^ Thus, the widespread shorthand for describing biological systems as nodes, connected by pointed arrows for activation and blunt arrows for repression, is potentially problematic. Unless the sequence of events is viewed dynamically, the descriptions of negative feedback are nonsensical. This should be familiar to anyone who has thought carefully about a gene network or biological process – indeed, since the 1960s researchers have developed logical frameworks for describing gene networks that explicitly consider the order of events in a network: the Boolean or logical network formalisms.^4^ Boolean networks avoid such contradictions with sequential time steps (synchronous or asynchronous) for switching states in feedback systems.^5^ This gives long sequences or histories of system states. Contrastingly, verbal paradoxes have no inherent time dimension (for a review, see^6^). The liar paradox analogy emphasises that commonly presented descriptions of biological interactions (A activates B. B inhibits A) are not understandable if viewed statically with dimensionless topology diagrams.

## An analogy between gene network motifs and the liar paradoxes

Network motifs are small patterns of interconnections that are embedded within large complex networks, from gene regulatory networks to the World Wide Web, and occur more frequently than one would expect by chance.^7^,^8^ Common motifs or topologies include: (i) negative-feedback loops, which reduce transcriptional noise^9^ and gene network response times^3^; (ii) positive-feedback loops, made of factors that activate themselves, which can lead to bistability^10^,^11^ and (iii) positive-negative or dual-negative topologies that can act as toggle switches,^12^,^13^ providing potential memory-coding units in cells.^14^ Many of these motifs or topologies involve feedback and can lead to oscillations or limit-cycle dynamic properties in biological systems.^15^–^17^

The role of biological feedback was neatly encapsulated in the conjectures of Thomas in the 1980s^5^,^18^,^19^: positive feedback is necessary for multiple steady states (multistationarity, *e.g*. a bistable network); negative feedback allows a wider range of attractors (recurrent gene activities). These attractors can be points (stable steady states), oscillators (limit cycles) or chaotic (non-linear feedback), depending on the specific interaction parameters involved. Thus, feedback is always modelled dynamically. The following sentence, however, has no time dimension:

[1] This statement is false.

This is a paradox because the statement implies the opposite of its own state (*i.e*. if it is true it is false and if it is false it is true). Considered instantaneously, there is a contradiction, because the two states cannot be compatible simultaneously. However, the statement contains an inherent feedback and when reading it to ourselves we tend to cycle between outcomes, inventing a time frame to give the opposing outputs (if it is true, *then* it is false, *then* it is true, *etc*.). By analogy, consider that if the opposite of ‘true’ is ‘false’, then similarly the opposite of ‘on’ is ‘off’ or, for an electronic logic gate, the opposite of output ‘1’ is ‘0’. Thus, this statement also implies the opposite tendency of its own state:

[2] This gene product represses its own production.

This can generate a similar ‘paradox’ if only the connections are considered instantaneously without dimensions: if a gene product exists, it switches itself off, therefore it no longer exists, therefore repression is removed, therefore residual activation dominates (through promoter leakiness or other activators), therefore the gene product exists. If it is on it is off and if it is off it is on (Table 1).

**Table 1:** Liar paradoxes and analogous gene network motifs

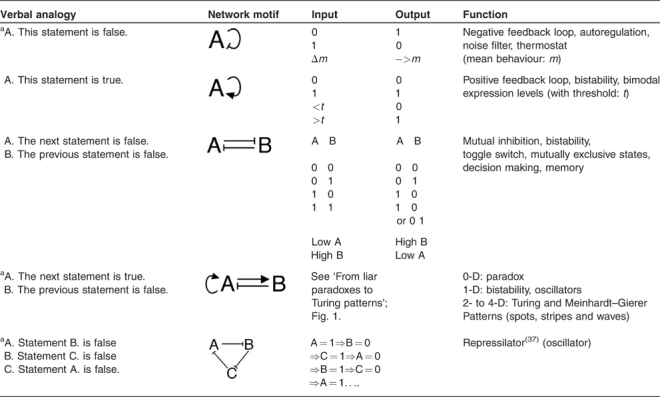

Only some networks are paradoxes (Marked:^a^). Others are self consistent if the history of the system is measured. Pointed arrows imply an activation interaction; blocked lines imply repression. For emphasis, networks are shown with all-or-nothing digital forms, with 1 and 0 for active or inactive states. Modelling the same network motif or ‘topology’ in different dimensions can give very different results: the penultimate example can go from paradox (dimensionless: 0-D), to oscillator (1-D), to stripe pattern (up to 4-D).

Although removing dimensions is obviously absurd, it stresses that there are opposite tendencies implicit within the system that can be resolved by adding the dimension of time, and by quantifying the control parameters. Opposing forces drive the system back towards an attractor, such as a mean or steady state, regardless of what perturbation is applied. Another analogy for this system is that it is like a thermostat, cycling between on and off, with stochastic fluctuations around its central position.^5^ Importantly, the central position is not necessarily off; the system can have a steady-state level, with turnover of components, as long as energy drives the system.

A consequence of this analogy is that it is meaningless to look for a true or false output of the circuit, the system must be considered over time in terms that take into account all the parameters involved in the network (production, degradation, repression, *etc*.^20^). So, if we want to understand sentence [1], perhaps we should attempt to measure the parameters controlling the negative feedback and ask *how false* is it over time?

## From paradoxes to Turing patterns

The liar analogy can be extended to other motifs as outlined in Table 1. For example, ‘This statement is true’ has a counterpart in the positive-feedback motif, which presents no logical problems in terms of paradoxes, but does have the property of bistability. Interestingly, the sentence itself could be considered to be ‘bistable’ with two states, true or false. Synthetic reconstruction of positive-feedback genetic circuits has revealed that they can indeed be bistable, especially when there is noise or fluctuation in gene expression, together with non-linear activation and appropriate protein degration.^10^,^11^

Another motif with bistability is the two-component system with mutual inhibition interactions^13^ (Table 1). This system is extremely common in biological networks and can be used at decision making branching points in biological pathways.^21^,^22^ For an intuitive understanding, imagine a domino balanced on its edge – pushing either side topples it, blocking the other side. In the same way, A:B mutual inhibition results in one side winning or losing the battle for expression and results in a robust mechanism for bistability (with appropriate repression, leakiness and component half-lives). The process can be summed up by a semantic analogy:

[3] A: The next statement is false. B: The previous statement is false.

This is not a paradox because either statement could be true; resolution depends on knowing the history of the system. Either A is true and B is false or *vice versa*; experimental measurements would reveal which is the case.

Biological implementations of mutual inhibition circuits are plentiful and they have even been studied in some elegant synthetic gene circuits where two transcription factors mutually repress each other.^23^,^24^ A neat example of a synthetic mutual inhibition gene circuit came from Ellis *et al*., who precisely characterised randomised promoter variants and then showed that the components could be used to engineer new networks with predictable properties.^23^ Mutually repressive TetR- and LacI-regulated promoter networks resulted in timers that could reliably flip states, to control the timing of a yeast sedimentation phenotype.

In the study of Kashiwagi *et al*.,^24^ it was found that *in vitro* ‘evolution’ could reliably select noise-adaptive states over non-adaptive states during mutual inhibition – the circuits could ‘flip’ to make the appropriate selections of which factor to select, even in the absence of signal transduction machinery. Cells will thus reliably select noise-adaptive states over non-adaptive states to render the latter less stable. Hence, from evolution's point of view, only instability may be stable.

Combining even numbers of negative interactions always results in multiple, mutually exclusive, stable states (*e.g*. ‘A inhibits B. B inhibits C. C inhibits D. D inhibits A.’ has two possible stable states if each factor can be on or off). On the other hand, combining odd numbers of negative interactions is rather more subtle, potentially leading to self-contradictions as in the dimensionless paradox:

[4] A: The next statement is true. B: The previous statement is false.

If A is true *then* B is false *then* A is false *then* B is true, *etc*. When adding the dimension of time, by analogy, this is very similar to the genetic clock proposed by Barkai and Leibler^25^ or the oscillator-toggle switch topology built by Atkinson *et al*.^12^:

[5] Autoactivator A makes repressor B. B represses A.

These network topologies are extremely common in biological networks and can give oscillations in time^14^,^26^–^28^ (Table 1). The verbal analogy gives an intuitive feel of why such systems can oscillate. However, it should be emphasised that each system is sensitive to its own parameters and that under certain conditions A or B can dominate (*e.g*. strong, long-lived A with weak, short-lived B).

So far we have only considered the dimension of time. Strikingly, when adding space, time and reaction diffusion, the same type of interaction gives a system that will be familiar to anyone interested in the mechanisms behind developmental pattern formation. The ‘A makes B. B inhibits A.’ interaction is a more general form of the Gierer and Meinhardt patterning mechanism (local activation and long-range lateral inhibition^29^,^30^). For example, consider:

[6] Autoactivator A diffuses slowly and activates B. Repressor B diffuses fast and represses A.

**Figure 1:**
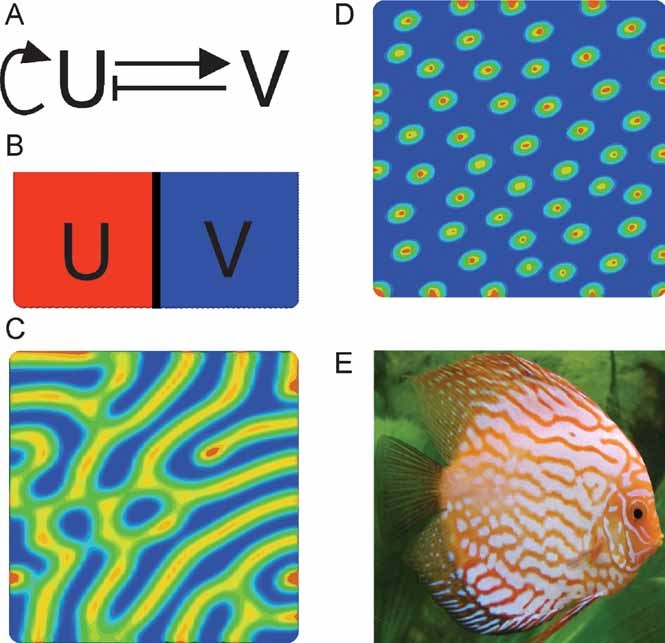
From liar paradoxes to Turing patterns. **A**: ‘The next statement is true. The previous statement is false’ is a zero-dimensional analogue of a stripe-pattern forming system, based on an activator U and inhibitor V (2-D space and 1-D time). Such systems rely on reaction and diffusion to make patterns (local autoactivation, long-range inhibition^29^,^56^). **B**: Rainbow colour coding shows local high expression of activator U (more red) and inhibitor V (more blue). With high autoactivation and weak repression, the activator dominates giving a uniform stability (red). With high transactivation and strong repression, the inhibitor dominates (blue). **C**: By balancing production, diffusion, reaction and degradation, spots, stripes or waves spontaneously emerge. **D**: This simulation has the same parameters as (C), except with a lower autocatalysis saturation constant, resulting in spots. **E**: The discus fish is thought to employ just such a body-patterning mechanism, based on reaction-diffusion. Images and calculations kindly provided by Luciano Marcon and James Sharpe. Discus image by Anka Zolnierzak

Although we are adding extra conditions to the simple topology in statement [4], the point that needs to be emphasised is that by adding more dimensions and reactions to the basic framework, intricate dynamic patterns can result (Fig. 1). Such reaction-diffusion patterns were originally discovered by Turing,^31^ and can result in spots,^32^ stripes^33^ or waves.^34^,^35^ Conversely, by removing dimensions, liar paradox topologies are obtained.

It should be pointed out that one does not need a Turing-type mechanism for ‘paradox-based’ pattern formation. Self-inhibitory dynamical behaviour in individual nuclei in a morphogenic fields is equally possible. An interesting example of this with just one diffusing morphogen was suggested by Lander.^36^ If a morphogen upregulates its own receptor, which is not only involved in signal transduction, but also ligand endocytosis, then two opposite effects occur at the same time: signalling increases close to the morphogen source (where ligand is not limiting), while it decreases further away (where ligand is limiting). Here, the two effects are not separated in time, but occur simultaneously at different locations in space.

## Odd numbers of negative interactions lead to oscillations

It would be possible to extend the verbal network analogy for many further examples, but we can finish with one final motif that nicely illustrates how combining simple connections leads to complicated outcomes. The repressilator^37^,^38^ is a network, built synthetically in *Escherichia coli*, that exhibits oscillatory behaviour through three consecutively repressive interactions (Table 1). The outcome of two consecutive negative interactions is to create a tendency towards positive expression, and thus there are transient activations in the system. If balanced with appropriate parameters and time delays between processes, this leads to oscillations. One can follow this intuitively by reading:

[7] A: Statement B is false. B: Statement C is false. C: Statement A is false.

Logically, if A is true, then B is false, then C is true, then A is false, then B is true, then C is false, then back to A is true. This cycle can be repeated endlessly, like the repressilator transcription–translation output.

We are used to thinking about systems such as transcription–translation networks in dynamic terms.^20^,^39^ However, the concept can be stretched to include problems not normally viewed as networks. These would include all kinds of metabolic and enzymatic processes with interdependent steps or processes.

For example, protein folding is usually regarded as a problem with a single optimal solution: the peptide chain folds through kinetic intermediates to find first local and then more global energy minima.^40^,^41^ However, protein folding and structure prediction are ‘difficult’ problems that cannot be solved analytically; much effort goes into simulations and approximations to find the best solution.^42^,^43^ The problem is computationally expensive because so many conformations are possible. Even here, it is likely that network concepts of circularity are an appropriate way of looking at such systems, as these can add a layer of useful information. In this way, take three hypothetical intramolecular protein–protein contacts – A, B and C – and consider them as a network. When position A is free it clashes with B, freeing it and fixing the position of A. When B is free it clashes with C, freeing it and fixing B. When C is free it clashes with A, freeing it and fixing C. As long as there is a supply and dissipation of free energy through the system, the result is a dynamic system with inherent contradictions that only makes sense if you look at it over both space and time. This can be extended to DNA or RNA base pairing, or even lipid association, with self-interaction forming order and asymmetries.

**Figure 2:**
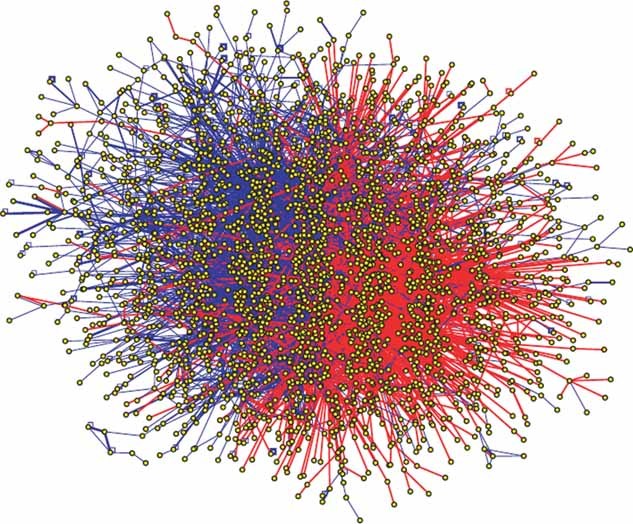
A ‘hairy monster’ diagram of the human protein–protein interactome.^46^ Health warning: static network descriptions may contain hidden behaviours. Image kindly provided by Marc Vidal. Reprinted by permission from Macmillan Publishers Ltd: Nature 2005. 437: 1173–1178, © 2005

Using *reductio ad absurdum* with the liar paradox, we see that static approaches are dangerous. Nowhere are static connections more apparent than in ‘interactome’ diagrams – descriptions of all the protein–protein interactions in a given genome. For example, Vidal and colleagues have produced wonderfully complicated interactome diagrams, for several organisms, that resemble ‘hairy monsters’^44^–^46^ (Fig. 2). We must all keep reminding ourselves that patterns of connections must be considered over multiple dimensions of time and space – or their feedbacks can imply different steady states, attractors or even self-contradictory liar paradoxes.

While the pioneers of interactomes are well-aware that defining system steady states will be required,^44^ elsewhere the message gets lost. For instance, p53 oscillations^26^,^47^ were long overlooked because of a static description being interpreted as a control switch to prevent expression levels from getting too high (*e.g*. p53 makes Mdm2. Mdm2 degrades p53, controlling p53 levels). As p53 is one of the best studied genes – over 20,000 PubMed papers contain p53 in the title – it took a surprisingly long time to explore the time-dimension explicity. However, should we really care whether p53 levels oscillate? Does this increase our biological insight into cancer? Perhaps the importance of such oscillations is not as immediately obvious to us as, say, the oscillations of the cell-division cycle, but there is a result here that may lead us to an important general principle in biological networks. There is an old saying (used to criticise generally false predictions) that goes, ‘Even a stopped clock is correct twice a day’. In biology, the converse may be more applicable: ‘An oscillating concentration gives you the correct concentration once or twice per cycle’. Thus, oscillating concentrations are more likely to give the correct ‘working’ amount at least some of the time, even if noise and other factors, keep disturbing the system. A thermostat or autoregulator is an oscillator with low amplitude, whereas circuits with larger amplitude may be more hit-and-miss – and will be correct some of the time.

Biological examples of oscillators based on negative feedback are numerous and include the SOS response^27^ or NF-κB signalling.^28^ If temporal oscillations have been frequently overlooked, multi-dimensional problems such as development would also benefit from a re-appraisal of the interactions between components. In summary, connection diagrams 48 give a comforting illusion of understanding a system, but we should all be wary of taking these networks at face value.

## Conclusions

This essay is primarily an alternative perspective on gene networks and does not aim to discuss liar paradoxes formally. For further reading, there is an accessible review by Dowden,^6^ which summarises the paradoxes and potential resolutions. Very briefly, these resolutions (amongst others) include suggesting the proposition is either meaningless,^1^,^49^ or neither true or false,^50^ or even that it is both true and false^6^ and as such occupies a unique position in language. More worryingly, Tarski was able to prove liar paradox inconsistency using an arithmetical argument that proved that true was not definable, and thus languages are either ‘semantically open’ or partly incoherent.^51^ What these solutions do not explore, however, is dimensionality or time and perhaps a more formal investigation of this possibility is required.

Although a source of delight to many, paradoxes have continued to trouble mathematicians and philosophers over the centuries.^52^ Russell was deeply shaken when a paradox concerning self-membership of groups threatened to undermine his belief that arithmetic was predicated on a hierarchy of sets.^53^ The ‘set that contains all sets that are not self-membered’ generates a by-now familiar cyclical eligibility – or non-eligibility – for being in its own set, depending on where you are in the logical loop.

Self-referential statements also profoundly influenced Gödel in his famous incompleteness theorems that destroyed the hope of a complete axiomatised system of mathematics.^54^ Gödel was able to generate statements of the form: ‘This sentence cannot be proved to be true’. If the statement is in fact true, then it must also be true that it is unprovable. If the statement is false, then it can be proved to be true, which is a clear contradiction. For an excellent discussion on this topic, the reader is referred to Hofstadter.^55^

In this essay, we have explored a path from philosophy to real-life gene circuits, and provided a brief overview of network motifs along the way. When faced with the liar paradox, Ludwig Wittgenstein is attributed with saying that one can only laugh because it resembles a joke. Clearly, he had a strange sense of humour but perhaps one should indeed not take these paradoxes too seriously. Nonetheless, as a final thought, I would like to point out that if we state now that everything presented in this paper is false, then we would have to be lying.
